# Consensus Recommendations on Training and Competing in the Heat

**DOI:** 10.1007/s40279-015-0343-6

**Published:** 2015-05-23

**Authors:** Sébastien Racinais, Juan-Manuel Alonso, Aaron J. Coutts, Andreas D. Flouris, Olivier Girard, José González-Alonso, Christophe Hausswirth, Ollie Jay, Jason K. W. Lee, Nigel Mitchell, George P. Nassis, Lars Nybo, Babette M. Pluim, Bart Roelands, Michael N. Sawka, Jonathan Wingo, Julien D. Périard

**Affiliations:** Aspetar, Qatar Orthopaedic and Sports Medicine Hospital, Research and Education Centre, PO Box 29222, Doha, Qatar; Sports Medicine Department, Aspetar Orthopaedic and Sports Medicine Hospital, Doha, Qatar; Medical and Anti-doping Commission, International Association of Athletics Federations (IAAF), Montecarlo, Monaco; Sport and Exercise Discipline Group, University of Technology Sydney (UTS), Lindfield, NSW Australia; FAME Laboratory, Department of Physical Education and Sport Science, University of Thessaly, Trikala, Greece; Department of Physiology, Faculty of Biology and Medicine, Institute of Sport Sciences, University of Lausanne (ISSUL), Lausanne, Switzerland; Department of Life Sciences, College of Health and Life Sciences, Centre for Sports Medicine and Human Performance, Brunel University London, Uxbridge, UK; Laboratory of Sport, Expertise and Performance, Research Department, French National Institute of Sport (INSEP), Paris, France; Discipline of Exercise and Sport Science, Faculty of Health Sciences, University of Sydney, Lidcombe, NSW Australia; Defence Medical and Environmental Research Institute, DSO National Laboratories, Singapore, Singapore; Yong Loo Lin School of Medicine, National University of Singapore, Singapore, Singapore; Lee Kong Chian School of Medicine, Nanyang Technological University, Singapore, Singapore; British Cycling and ‘Sky Pro Cycling’, National Cycling Centre, Manchester, UK; National Sports Medicine Programme, Excellence in Football Project, Aspetar, Qatar Orthopaedic and Sports Medicine Hospital, Doha, Qatar; Department of Nutrition, Exercise and Sport, Section of Human Physiology, University of Copenhagen, Copenhagen, Denmark; Medical Department, Royal Netherlands Lawn Tennis Association (KNLTB), Amersfoort, The Netherlands; Department of Human Physiology, Vrije Universiteit Brussel, Brussels, Belgium; School of Applied Physiology, College of Science, Georgia Institute of Technology, Atlanta, GA USA; Department of Kinesiology, University of Alabama, Tuscaloosa, AL USA

## Abstract

Exercising in the heat induces thermoregulatory and other physiological strain that can lead to impairments in endurance exercise capacity. The purpose of this consensus statement is to provide up-to-date recommendations to optimize performance during sporting activities undertaken in hot ambient conditions. The most important intervention one can adopt to reduce physiological strain and optimize performance is to heat acclimatize. Heat acclimatization should comprise repeated exercise–heat exposures over 1–2 weeks. In addition, athletes should initiate competition and training in an euhydrated state and minimize dehydration during exercise. Following the development of commercial cooling systems (e.g., cooling vests), athletes can implement cooling strategies to facilitate heat loss or increase heat storage capacity before training or competing in the heat. Moreover, event organizers should plan for large shaded areas, along with cooling and rehydration facilities, and schedule events in accordance with minimizing the health risks of athletes, especially in mass participation events and during the first hot days of the year. Following the recent examples of the 2008 Olympics and the 2014 FIFA World Cup, sport governing bodies should consider allowing additional (or longer) recovery periods between and during events for hydration and body cooling opportunities when competitions are held in the heat.

## Aim and Scope

Most of the major international sporting events such as the Summer Olympics, the FIFA World Cup, and the Tour de France—i.e., the three most popular events in terms of television audience worldwide—take place during the summer months of the northern hemisphere, and often in hot ambient conditions. On 23 and 24 March 2014, a panel of experts reviewed and discussed the specificities of training and competing in the heat during a topical conference held at Aspetar Orthopaedic and Sports Medicine Hospital in Doha, Qatar. The conference ended with a round-table discussion, which has resulted in this consensus statement.

This document is intended to provide up-to-date recommendations regarding the optimization of exercise capacity during sporting activities in hot ambient conditions. Given that the performance of short-duration activities (e.g., jumping and sprinting) is at most marginally influenced, or can even be improved, in hot ambient conditions [1], but that prolonged exercise capacity is significantly impaired [2], the recommendations provided in this consensus statement focus mainly on prolonged sporting events. For additional information, the reader is referred to the supplement issue, *Training and Competing in the Heat*, published in the *Scandinavian Journal of Medicine and Science in Sports*, which includes targeted reviews and original manuscripts [3].

## Background

When exercising in the heat, skin blood flow and the sweat rate increase to allow for heat dissipation to the surrounding environment. These thermoregulatory adjustments, however, increase physiological strain and may lead to dehydration during prolonged exercise. Heat stress alone will impair aerobic performance when hyperthermia occurs [2, 4–6]. Consequently, athletes perform endurance, racket, or team sports events in the heat at a lower work rate than in temperate environments [7–12]. In addition, dehydration during exercise in the heat exacerbates thermal and cardiovascular strain [13–18] and further impairs aerobic performance [2, 17, 19]. This document contains recommendations and strategies to adopt in order to sustain/enhance performance during training and competition in the heat, as well as to minimize the risk of exertional heat illness. As presented in Sect. 3, the most important intervention one can adopt to reduce physiological strain and optimize performance is to heat acclimatize. Given that dehydration can impair physical performance and exacerbate exercise-induced heat strain, Sect. 4 of the consensus statement provides recommendations regarding hydration. Section 5 highlights the avenues through which it is possible to decrease core and skin temperatures before and during exercise via the application of cold garments to the skin such as ice packs, cold towels and cooling vests, as well as through cold water immersion (CWI) or ice slurry ingestion.

Given the lack of data from real competitions, the International Olympic Committee (IOC) recently highlighted the necessity for sports federations, team doctors, and researchers to collaborate in obtaining data on the specific population of elite athletes exercising in challenging environments [20]. Several international sporting federations, such as FIFA (Fédération Internationale de Football Association), FINA (Fédération Internationale de Natation), FIVB (Fédération Internationale de Volleyball), IAAF (International Association of Athletics Federations), and ITF (International Tennis Federation), have responded to this challenge by initiating a surveillance system to assess environmental conditions during competition, along with their adverse outcomes [12, 21–23]. A number of sporting federations have also edited their guidelines to further reduce the risks of exertional heat illness. These guidelines are reviewed in Sect. 6 of this consensus statement. Recommendations are offered to event organizers and sporting bodies on how to best protect the health of the athlete and sustain/enhance performance during events in the heat.

## Heat Acclimatization

Although regular exercise in temperate conditions elicits partial heat acclimatization [24], it cannot replace the benefits induced by consecutive days of training in the heat [24–27]. Heat acclimatization improves thermal comfort and submaximal as well as maximal aerobic exercise performance in warm–hot conditions [11, 28, 29]. The benefits of heat acclimatization are achieved via increased sweating and skin blood flow responses, plasma volume expansion, and, hence, improved cardiovascular stability (i.e., better ability to sustain blood pressure and cardiac output) and fluid-electrolyte balance [19, 30, 31]. Exercise–heat acclimatization is therefore essential for athletes preparing for competitions in warm–hot environments [30]. This section describes how to practically implement heat acclimatization protocols and optimize the benefits in athletes.

### Induction of Acclimatization

#### Duration

Most adaptations (i.e., decreases in heart rate, skin and rectal temperature, increases in sweat rate, and work capacity) develop within the first week of heat acclimatization and more slowly in the subsequent 2 weeks [32–34]. Adaptations develop more quickly in highly trained athletes (up to half the time) compared with untrained individuals [24, 35]. Consequently, athletes benefit from only a few days of heat acclimatization [36–38], but may require 6–10 days to achieve near-complete cardiovascular and sudomotor adaptations [28, 29, 39], and as such 2 weeks to optimize aerobic performance (i.e., cycling time trial) in hot ambient conditions [11].

#### Training

The principle underlying any heat acclimatization protocol is an increase in body (core and skin) temperature to induce profuse sweating and increase skin blood flow [19, 30]. Repeated heat–exercise training for 100 min was originally shown to be efficient at inducing such responses [40]. Reportedly, exercising daily to exhaustion at 60 % of maximal oxygen uptake (*V*O_2max_) in hot ambient conditions [40 °C, 10 % relative humidity (RH)] for 9–12 consecutive days increases exercise capacity from 48 to 80 min [28]. Ultimately, the magnitude of adaptation depends on the intensity, duration, frequency, and number of heat exposures [30, 31]. For example, Houmard et al. [41] reported similar physiological adaptations following moderate-intensity short-duration (30–35 min, 75 % *V*O_2max_) and low-intensity long-duration (60 min, 50 % *V*O_2max_) exercise.

As acclimatization develops, constant workload exercise protocols may result in a progressively lower training stimulus (i.e., decreases in relative exercise intensity). In turn, this may limit the magnitude of adaptation if the duration and/or the intensity of the heat–exercise training sessions are not increased accordingly [42]. When possible, an isothermic protocol (e.g., controlled hyperthermia to a core temperature of at least 38.5 °C) can be implemented to optimize the adaptations [43, 44]. However, isothermic protocols may require greater control and the use of artificial laboratory conditions, which could limit their practicality in the field. Alternatively, it has recently been proposed to utilize a controlled intensity regimen based on heart rate to account for the need to increase absolute intensity and maintain a similar relative intensity throughout the acclimatization process [31]. Lastly, athletes can adapt by training outdoors in the heat (i.e., acclimatization) using self-paced exercise or by maintaining their regular training regimen. The efficacy of this practice has been demonstrated with team-sport athletes [45, 46], without interfering with their training regimen.

#### Environment

Heat acclimatization in dry heat improves exercise in humid heat [47, 48], and vice versa [49]. However, acclimatization in humid heat evokes higher skin temperatures and circulatory adaptations than in dry heat, potentially increasing maximum skin wettedness and therefore the maximum rate of evaporative heat loss from the skin [30, 31, 50]. Although scientific support for this practice is still lacking, it may potentially be beneficial for athletes to train in humid heat at the end of their acclimatization sessions to dry heat to further stress the cardiovascular and thermoregulatory systems. Nevertheless, despite some transfer between environments, other adaptations might be specific to the climate (desert or tropic) and physical activity level [51]. Consequently, it is recommended that athletes predominantly acclimatize to the environment in which they will compete.

Athletes who do not have the possibility to travel to naturally hot ambient conditions (so-called ‘acclimatization’) can train in an artificially hot indoor environment (so-called ‘acclimation’). However, whilst acclimation and acclimatization share similar physiological adaptations, training outdoors is more specific to the competition setting as it allows athletes to experience the exact nature of the heat stress [52–54].

### Decay and Periodization of Short-Term Acclimatization

Heat adaptations decay at different rates, with the fastest adaptations also decaying more rapidly [35]. However, the rate of decay of heat acclimatization is generally slower than its induction, allowing maintenance of the majority of benefits (e.g., heart rate, core temperature) for 2–4 weeks [34, 55–58]. Moreover, during this period, individuals (re)acclimatize faster than during the first acclimatization period [57] (Table 1). These studies are, however, mainly based on physiological markers of heat acclimatization and the decay in competitive sporting performance remains to be clarified.

**Table 1:** Examples of heat-acclimatization strategies

	Objective	Duration	Period	Content	Environment
Pre-/in-season training camp	Enhance/boost the training stimulus	1–2 weeks	Pre-season or in-season	Regular or additional training (75–90 min/day) to increase body temperature and induce profuse sweating	Natural or artificial heat stress
Target competition preparatory camp	Optimize future re-acclimatization and evaluate individual responses in the heat	2 weeks	1 month before competing in the heat	Regular or additional training, simulated competition, and heat response test	Equivalent to or more stressful than target competition
Target competition final camp	Optimize performance in the heat	1–2 weeks, depending on results of preparatory camp	Just before the competition	Pre-competition training	Same as competition

### Individualized Heat Acclimatization

Heat acclimatization clearly attenuates physiological strain [59, 60]. However, individual acclimatization responses may differ and should be monitored using simple indices, such as the lessened heart rate increase during a standard sub-maximal exercise bout [33, 61–63]. Other more difficult and likely less sensitive markers for monitoring heat acclimatization include sweat rate and sodium content [64], core temperature [33], and plasma volume [65]. The role of plasma volume expansion in heat acclimatization remains debated as an artificial increase in plasma volume does not appear to improve thermoregulatory function [66, 67], but the changes in hematocrit during a heat-response test following short-term acclimatization correlate to individual physical performance [45, 46]. This suggests that plasma volume changes might represent a valuable indicator, even if it is probably not the physiological mechanism improving exercise capacity in the heat. Importantly, measures in a temperate environment cannot be used as a substitute to a test in hot ambient temperatures [45, 46, 68].

As with its induction, heat acclimatization decay also varies between individuals [32]. It is therefore recommended that athletes undergo an acclimatization procedure months before an important event in the heat to determine their individual rate of adaptation and decay [20, 45] (Table 1).

### Heat Acclimatization as a Training Stimulus

Several recent laboratory or uncontrolled field studies have reported physical performance improvement in temperate environments following training in the heat [29, 46, 62, 69, 70]. Athletes might therefore consider using training camps in hot ambient conditions to improve physical performance both in-season [62] and pre-season [46] (Table 1). Bearing in mind that training quality should not be compromised, the athletes benefiting the most from this might be experienced athletes requiring a novel training stimulus [46], whereas the benefit for highly trained athletes with limited thermoregulatory requirement (e.g., cycling in cold environments) might be more circumstantial [71].

### Summary of the Main Recommendations for Heat Acclimatization

- Athletes planning to compete in hot ambient conditions should heat acclimatize (i.e., repeated training in the heat) to obtain biological adaptations lowering physiological strain and improving exercise capacity in the heat.
- Heat acclimatization sessions should last at least 60 min per day and induce an increase in body core and skin temperatures, as well as stimulate sweating.
- Athletes should train in the same environment as the competition venue, or, if not possible, train indoors in a hot room.
- Early adaptations are obtained within the first few days, but the main physiological adaptations are not complete until ~1 week. Ideally the heat acclimatization period should last 2 weeks in order to maximize all benefits.

## Hydration

The development of hyperthermia during exercise in hot ambient conditions is associated with a rise in sweat rate, which can lead to progressive dehydration if fluid losses are not minimized by increasing fluid consumption. Exercise-induced dehydration, leading to a hypohydrated state, is associated with a decrease in plasma volume and an increase in plasma osmolality that are proportional to the reduction in total body water [19]. The increase in the core temperature threshold for vasodilation and sweating at the onset of exercise is closely linked to the ensuing hyperosmolality and hypovolemia [72, 73]. During exercise, plasma hyperosmolality reduces the sweat rate for any given core temperature and decreases evaporative heat loss [74]. In addition, dehydration decreases cardiac filling and challenges blood pressure regulation [75–77]. The rate of heat storage and cardiovascular strain is therefore exacerbated and the capacity to tolerate exercise in the heat is reduced [78–80].

Despite decades of studies in this area [81], the notion that dehydration impairs aerobic performance in sport settings is not universally accepted and there seems to be a two-sided polarized debate [82–84]. Numerous studies report that dehydration impairs aerobic performance in conditions where exercise is performed in warm–hot environments and body water deficits exceed at least ~2 % of body mass [13, 49, 81, 85–90]. On the other hand, some recent studies suggest that dehydration up to 4 % of body mass does not alter cycling performance under ecologically valid conditions [82, 83, 91]. However, these results must be interpreted in context; that is, in well-trained male cyclists typically exercising for 60 min in ambient conditions up to 33 °C and 60 % RH and starting exercise in an euhydrated state. Nonetheless, some have advanced the idea that the detrimental consequences of dehydration have been overemphasized by sports beverage companies [92]. As such, it has been argued that athletes should drink to thirst [82, 83, 91]. However, many studies (often conducted prior to the creation and marketing of ‘sport-drinks’) have repeatedly observed that drinking to thirst often results in body water deficits that may exceed 2–3 % of body mass when sweat rates are high and exercise is performed in warm–hot environments [13, 47, 49, 93–98]. Ultimately, drinking to thirst may be appropriate in many settings, but not in circumstances where severe dehydration is expected (e.g., an Ironman triathlon) [84].

In competition settings, hydration is dependent on several factors, including fluid availability and the specificities of the events. For example, whilst tennis players have regular access to fluids due to the frequency of breaks in a match, other athletes such as marathon runners have less opportunity to rehydrate. There are also differences among competitors. Whereas the fastest marathon runners do not consume a large volume of fluids and become dehydrated during the race, some slower runners may, conversely, overhydrate [99], with an associated risk of ‘water intoxication’ (i.e., hyponatremia) [100]. The predisposing factors related to developing hyponatremia during a marathon include substantial weight gain, a racing time above 4 h, female sex, and low body mass index [101, 102]. Consequently, although the recommendations below for competitive athletes explain how to minimize the impairment in performance associated with significant dehydration and body mass loss (i.e., ≥2 %), recreational athletes involved in prolonged exercise should be cautious not to overhydrate during exercise.

### Pre-exercise Hydration

Resting and well-fed humans are generally well-hydrated [103] and the typical variance in day-to-day total body water fluctuates from 0.2 to 0.7 % of body mass [93, 104]. When exposed to heat stress in the days preceding competition, it may, however, be advisable to remind athletes to drink sufficiently and replace electrolyte losses to ensure that euhydration is maintained. Generally, drinking 6 mL of water per kg of body mass during this period every 2–3 h, as well as 2–3 h before training or competition in the heat is advisable.

There are several methods available to evaluate hydration status, each one having limitations depending upon how and when the fluids are lost [105, 106]. The most widely accepted and recommended methods include monitoring body mass changes, and measuring plasma osmolality and urine-specific gravity. Based on these methods, one is considered euhydrated if daily body mass changes remain <1 %, plasma osmolality is <290 mmol/kg, and urine-specific gravity is <1.020. These techniques can be implemented during intermittent competitions lasting for several days (e.g., a cycling stage race, tennis/team sports tournament) to monitor hydration status. Establishing baseline body mass is important as daily variations may occur. It is best achieved by measuring post-void nude body mass in the morning on consecutive days after consuming 1–2 L of fluid the prior evening [81]. Moreover, since exercise, diet, and prior drinking influence urine concentration measurements, first morning urine is the preferred assessment timepoint to evaluate hydration status [81]. If first morning urine cannot be obtained, urine collection should be preceded by several hours of minimal physical activity, fluid consumption, and eating.

### Exercise Hydration

Sweat rates during exercise in the heat vary dramatically depending upon the metabolic rate, environmental conditions, and heat acclimatization status [107]. While values ranging from 1.0 to 1.5 L/h are common for athletes performing vigorous exercise in hot environments, certain individuals can exceed 2.5 L/h [108–111]. Over the last several decades, mathematical models have been developed to provide sweat loss predictions over a broad range of conditions [112–117]. Whilst these have proven useful in public health, military, and occupational and sports medicine settings, these models require further refinement and individualization to athletic populations, especially elite athletes.

The main electrolyte lost in sweat is sodium (20–70 mEq/L) [118, 119] and supplementation during exercise is often required for heavy and ‘salty’ sweaters to maintain plasma sodium balance. Heavy sweaters may also deliberately increase sodium (i.e., salt) intake prior to and following hot-weather training and competition to maintain sodium balance (e.g., 3.0 g of salt added to 0.5 L of a carbohydrate–electrolyte drink). To this effect, the Institute of Medicine [103] has highlighted that public health recommendations regarding sodium ingestion do not apply to individuals who lose large volumes of sodium in sweat, such as athletes training or competing in the heat. A salt intake that would not compensate sweat sodium losses would result in a sodium deficit that might prompt muscle cramping when reaching 20–30 % of the exchangeable sodium pool [120]. During exercise lasting longer than 1 h, athletes should therefore aim to consume a solution containing 0.5–0.7 g/L of sodium [121–123]. In athletes experiencing muscle cramping, it is recommended to increase the sodium supplementation to 1.5 g/L of fluid [124]. Athletes should also aim to include 30–60 g/h of carbohydrates in their hydration regimen for exercise lasting longer than 1 h [122], and up to 90 g/h for events lasting over 2.5 h [125]. This can be achieved through a combination of fluids and solid foods.

### Post-exercise Rehydration

Following training or competing in the heat, rehydration is particularly important to optimize recovery. If a fluid deficit needs to be urgently replenished, it is suggested to replace 150 % of body mass losses within 1 h following the cessation of exercise [123, 126], including electrolytes to maintain total body water. From a practical perspective, this may not be achievable for all athletes for various reasons (e.g., time, gastrointestinal discomfort). Thus, it is more realistic to replace 100–120 % of body mass losses. The preferred method of rehydration is through the consumption of fluids with foods (e.g., including salty food).

Given that exercise in the heat increases carbohydrate metabolism [127, 128], endurance athletes should ensure that not only water and sodium losses are replenished, but carbohydrates stores as well [129]. To ensure the highest rates of muscle glycogen resynthesis, carbohydrates should be consumed during the first hour after exercise [130]. Moreover, a drink containing protein (e.g., milk) might allow better restoration of fluid balance after exercise than a standard carbohydrate–electrolyte sports drink [131]. Combining protein (0.2–0.4 g/kg/h) with carbohydrate (0.8 g/kg/h) has also been reported to maximize protein synthesis rates [132]. Therefore, athletes should consider consuming drinks such as chocolate milk, which has a carbohydrate-to-protein ratio of 4:1, as well as sodium following exercise [133].

### Summary of the Main Recommendations for Hydration

- Before training and competition in the heat, athletes should drink 6 mL of fluid per kg of body mass every 2–3 h, in order to start exercise euhydrated.
- During intense prolonged exercise in the heat, body water mass losses should be minimized (without increasing body weight) to reduce physiological strain and help to preserve optimal performance.
- Athletes training in the heat have higher daily sodium (i.e., salt) requirements than the general population. Sodium supplementation might also be required during exercise.
- For competitions lasting several days (e.g., a cycling stage race, tennis/team sports tournament), simple monitoring techniques such as daily morning body mass and urine specific gravity can provide useful insights into the hydration state of the athlete.
- Adequately rehydrating after exercise–heat stress by providing plenty of fluids with meals is essential. If aggressive and rapid replenishment is needed, then consuming fluids and electrolytes to offset 100–150 % of body mass losses will allow for adequate rehydration.
- Recovery hydration regimens should include sodium, carbohydrates, and protein.

## Cooling Strategies

Skin cooling will reduce cardiovascular strain during exercise in the heat, while whole-body cooling can reduce organ and skeletal muscle temperatures. Several studies carried out in controlled laboratory conditions (e.g., uncompensable heat stress), in many cases with or without reduced fanning during exercise, have reported that pre-cooling can improve endurance [134–140] and high-intensity [141] and intermittent- or repeated-sprints exercise performance [142–145]. However, several other studies reported no performance benefits of pre-cooling on intermittent- or repeated-sprints exercise performance in the heat [142, 146–148]. Whole-body cooling (including cooling of the exercising muscles) may even be detrimental to performance during a single sprint or the first few repetitions of an effort involving multiple sprints [149, 150].

Therefore, whereas several reviews concluded that cooling interventions can increase prolonged exercise capacity in hot conditions [151–158], it has to be acknowledged that most laboratory-based pre-cooling studies might have overestimated the effect of pre-cooling as compared to an outdoor situation with airflow [159], or do not account for the need to warm-up before competing. As a consequence, the effectiveness of cooling in competitive settings remains equivocal and the recommendations below are limited to prolonged exercise in hot ambient conditions with no or limited air movement.

### Cold Water Immersion

A range of CWI protocols are available (as discussed in recent reviews [156, 160–162]), but the most common techniques are whole-body CWI for ~30 min at a water temperature of 22−30 °C or body segment (e.g., legs) immersion at lower temperatures (10−18 °C) [156]. However, cooling of the legs/muscles will decrease nerve conduction and muscle contraction velocities [1] and athletes might therefore need to re-warm-up before competition. Consequently, other techniques involving cooling garments have been developed to selectively cool the torso, which may prevent the excessive cooling of active muscles whilst reducing overall thermal and cardiovascular strain.

### Cooling Garments

Building on the early practice of using iced towels for cooling purposes, several manufacturers have designed ice-cooling jackets to cool athletes before or during exercise [137, 142, 163, 164]. The decrease in core temperature is smaller with a cooling vest than with CWI or mixed–cooling methods [158], but cooling garments present the advantage of lowering skin temperature and thus reducing cardiovascular strain and eventually heat storage [165]. Cooling garments are practical in reducing skin temperature without reducing muscle temperature, and athletes can wear them during warm-up or recovery breaks.

### Cold Fluid Ingestion

Cold fluids can potentially enhance endurance performance when ingested before [166, 167], but not during [168, 169], exercise. Indeed, it is suggested that a downside of ingesting cold fluids during exercise might be a reduction in sweating and therefore skin surface evaporation [170], due to the activation of thermoreceptors probably located in the abdominal area [171].

### Ice-Slurry Beverages

Based on the theory of enthalpy, ice requires substantially more heat energy (334 J/g) to cause a phase change from solid to liquid (at 0 °C) than the energy required to increase the temperature of water (4 J/g/°C). As such, ice slurry may be more efficient than cold water ingestion in cooling athletes. However, it is not yet clear if the proportional reduction in sweating observed with the ingestion of cold water during exercise [170] occurs with ice slurry ingestion. Several recent reports support the consumption of an ice-slurry beverage since performance during endurance or intermittent-sprint exercise is improved following the ingestion of an ice-slurry beverage (~1 L crushed ice at ≤4 °C) either prior to [140, 172, 173] or during exercise [174], but no benefit was evident when consumed during the recovery period between two exercise bouts in another study [175]. Consequently, ingestion of ice slurry may be a practical complement or alternative to external cooling methods [155], but more studies are still required during actual outdoors competitions.

### Mixed-Methods Cooling Strategies

Combining techniques (i.e., using both external and internal cooling strategies) has a higher cooling capacity than the same techniques used in isolation, allowing for greater benefit on exercise performance [158]. Indeed, mixed methods have proven beneficial when applied to professional football players during competition in the tropics [176], lacrosse players training in hot environments [177], and cyclists simulating a competition in a laboratory [139]. In a sporting context, this can be achieved by combining simple strategies, such as the ingestion of ice slurry, wearing cooling vests, and providing fanning.

### Cooling to Improve Performance Between Subsequent Bouts of Exercise

There is evidence supporting the use of CWI (5–12 min in 14 °C water) during the recovery period (e.g., 15 min) separating intense exercise bouts in the heat to improve subsequent performance [178, 179]. The benefits of this practice would relate to a redistribution of the blood flow, probably from the skin to the central circulation [180], as well as a psychological (i.e., placebo) effect [181]. In terms of internal cooling, the ingestion of cold water [182] or ice slurry [175] during the recovery period might attenuate heat strain in the second bout of work, but not necessarily significantly improve performance [175]. Together, these studies suggest that cooling might help recovery from intense exercise in uncompensable laboratory heat stress and, in some cases, might improve performance in subsequent intense exercise bouts. The effects of aggressive cooling versus simply resting in the prevailing hot ambient conditions, or in cooler conditions, remains to be validated in a competition setting (e.g., half time in team sports).

### Summary of the Main Recommendations for Cooling

- Cooling methods include external (e.g., application of iced garments, towels, water immersion, or fanning) and internal methods (e.g., ingestion of cold fluids or ice slurry).
- Pre-cooling may benefit sporting activities involving sustained exercise (e.g., middle- and long-distance running, cycling, tennis, and team sports) in warm–hot environments. Internal methods (i.e., ice slurry) can be used during exercise, whereas tennis and team sport athletes can also implement mixed cooling methods during breaks.
- Such practice may not be viable for explosive or shorter-duration events (e.g., sprinting, jumping, throwing) conducted in similar conditions.
- A practical approach in hot–humid environments might be the use of fans and commercially available ice-cooling vests, which can provide effective cooling without impairing muscle temperature. In any case, cooling methods should be tested and individualized during training to minimize disruption to the athlete.

## Recommendations for Event Organizers

The most common set of recommendations followed by event organizers to reschedule or cancel an event is based on the Wet-Bulb Globe Temperature (WBGT) index empirically developed by the US military, popularized in sports medicine by the American College of Sports Medicine [183] and adopted by various sporting federations (Table 2). However, WBGT might underestimate the heat-stress risk when sweat evaporation is restricted (i.e., high humidity and/or low air movement) [184]. Thus, corrected recommendations have been proposed [185] (Table 3). Moreover, the WBGT is a climatic index and does not account for metabolic heat production or clothing and therefore cannot predict heat dissipation [19]. Therefore, the recommendations below provide guidelines for various sporting activities rather than fixed cut-offs based on the WBGT index.

**Table 2:** Examples of recommended actions by various sporting governing bodies based on the Wet-Bulb Globe Temperature index

WBGT (^o^C)	Organization	Athlete concerned	Recommendation
32.3	ACSM	Acclimatized, fit, and low-risk individuals	Participation cut-off
32.2	ITF	Junior and wheelchair tennis players	Immediate suspension of play
32.2	WTA	Female tennis players	Immediate suspension of play
32.0	FIFA	Football players	Additional cooling break at 30 and 75 min
30.1	ACSM	Non-acclimatized, unfit, and high-risk individuals	Participation cut-off
30.1	ITF–WTA	Junior and female tennis players	10-min break between 2nd and 3rd set
30.1	ITF	Wheelchair tennis players	Suspension of play at the end of the set in progress
28.0	ITF	Wheelchair tennis players	15-min break between 2nd and 3rd set
28.0	Australian Open	Tennis players	10-min break between 2nd and 3rd set
21.0	Marathon in northern latitudes	Runners in mass participation events	Cancel marathon

Data from ACSM [183], Roberts [192], and from the following websites: http://www.fifa.com/aboutfifa/footballdevelopment/medical/playershealth/risks/heat.html, http://www.itftennis.com/media/194281/194281.pdf, http://www.itftennis.com/media/195690/195690.pdf, http://www.wtatennis.com/SEWTATour-Archive/Archive/AboutTheTour/rules2015.pdf, and http://www.ausopen.com/en_AU/event_guide/a_z_guide.html

*ACSM* American College of Sports Medicine, *FIFA* Fédération Internationale de Football Association*, ITF* International Tennis Federation, *WBGT* Wet Bulb Globe Temperature, *WTA* Women’s Tennis Association

**Table 3:** Corrected estimation of the risk of exertional heat illness based on the Wet-Bulb Globe Temperature (WBGT) index, taking into account that WBGT underestimates heat stress under high humidity

Estimated risk	WBGT (^o^C)	Relative humidity (%)
Moderate	24	50
Moderate	20	75
Moderate	18	100
High	28	50
High	26	75
High	24	100
Excessive	33	50
Excessive	29	75
Excessive	28	100

Adapted from the categories proposed by Gonzalez [185] to estimate the risk of exertional heat illness during a marathon

### Cancelling an Event or Implementing Countermeasures?

Further to appropriate scheduling of any event with regards to expected environmental conditions, protecting athlete health might require stopping competition when combined exogenous and endogenous heat loads cannot be physiologically compensated. The environmental conditions in which the limit of compensation is exceeded depends on several factors, such as metabolic heat production (depending on workload and efficiency/economy), athlete morphology (e.g., body surface area to mass ratio), acclimatization state (e.g., sweat rate), and clothing. It is therefore problematic to establish universal cut-off values across different sporting disciplines. Environmental indices should be viewed as recommendations for event organizers to implement preventive countermeasures to offset the potential risk of heat illness. The recommended countermeasures include adapting the rules and regulations with regards to cooling breaks and the availability of fluids (time and locations), as well as providing active cooling during rest periods. It is also recommended that medical response protocols and facilities to deal with cases of exertional heat illnesses be in place.

### Specificity of the Recommendations

#### Differences Among Sports

Hot ambient conditions impair endurance exercise such as marathon running [7], but potentially improve short-duration events such as jumping or sprinting [1]. In many sports, athletes adapt their activity according to the environmental conditions. For example, compared to cooler conditions, football players decrease the total distance covered or the distance covered at high intensity during a game, but maintain their sprinting activity/ability [9, 12, 186], while tennis players reduce point duration [8] or increase the time between points [10] when competing in the heat (WBGT ~34 °C). Event organizers and international federations should therefore acknowledge and support such behavioral thermoregulatory strategies by adapting the rules and refereeing accordingly.

#### Differences Among Individuals Within a Given Sport

When comparing two triathlon races held in Melbourne (VIC, Australia), in similar environmental conditions (i.e., WBGT raising from 22 to 27 °C during each race), 2 months apart, Gosling et al. [187] observed 15 cases of exertional heat illness (including three heat strokes) in the first race that was held in unseasonably hot weather at the start of summer, but no cases in the second race. This suggests that the risk of heat illness was increased in competitors who were presumably not seasonally heat acclimatized [187] and supports many earlier studies regarding the increased risk of heat illness in early summer or with hot weather spikes [188]. Nevertheless, exertional heat stroke can occur in individuals who are well-acclimatized and have performed similar activities several times before, as they may suffer from prior viral infection or similar ailment [19]. In one of the very few epidemiological studies linking WBGT to illness in athletes, Bahr and Reeser [22] investigated 48 beach volleyball matches (World Tour and World Championships) over 3 years. They reported only one case of a heat-related medical forfeit, which was related to an athlete with compromised fluid balance due to a 3-day period of acute gastroenteritis [22]. Moreover, whilst healthy runners can also finish a half-marathon in warm and humid environments without developing heat illness [189], exertional heat stroke has been shown to occur during a cool-weather marathon in a runner recovering from a viral infection [190].

In fact, prior viral infection is emerging as a potentially important risk factor for heat injury/stroke [19, 191]. Event organizers should therefore pay particular medical attention to all populations potentially at a greater risk, including participants currently sick or recovering from a recent infection, those with diarrhea, recently vaccinated, with limited heat dissipation capacity due to medical conditions (e.g., Paralympic athletes), or individuals involved in sports with rules restricting heat dissipation capacity (e.g., protective clothing/equipment). Unacclimatized participants are also to be considered at risk. Although it is impractical to screen every athlete during large events, organizers are encouraged to provide information, possibly in registration kits, advising all athletes of the risk associated with participation under various potential compromised states and suggesting countermeasures.

### Summary of the Main Recommendations for Event Organizers

- The WBGT is an environmental heat stress index and not a representation of human heat strain. It is therefore difficult to establish absolute participation cut-off values across sports for different athletes and we rather recommend implementing preventive countermeasures or evaluating the specific demands of the sport when preparing extreme heat policies.
- Countermeasures include scheduling the start time of events based on weather patterns, adapting the rules and refereeing to allow extra breaks or longer recovery periods, developing a medical response protocol and cooling facilities.
- Event organizers should pay particular attention to all ‘at risk’ populations. Given that unacclimatized participants (mainly in mass participation events) are at a higher risk for heat illness, organizers should properly advise participants of the risk associated with participation, or consider canceling an event in the case of unexpected or unseasonably hot weather.

## Conclusion

Our current knowledge on heat stress is mainly derived from military and occupational research fields, while the input from sport sciences is more recent. Based on this literature, athletes should train for at least 1 week and ideally 2 weeks to acclimatize using a comparable degree of heat stress as the target competition. They should also be cautious to undertake exercise in an euhydrated state and minimize body water deficits (as monitored by body mass losses) through proper rehydration during exercise. They can also implement specific countermeasures (e.g., cooling methods) to reduce heat storage and physiological strain during competition and training, especially when the environmental conditions are uncompensable. Event organizers and sports governing bodies can support athletes by allowing additional (or longer) recovery periods for enhanced hydration and cooling opportunities during competitions in the heat.

## References

[1] Racinais S, Oksa J (2010). Temperature and neuromuscular function. Scand J Med Sci Sports..

[2] Nybo L, Rasmussen P, Sawka MN (2014). Performance in the heat—physiological factors of importance for hyperthermia-induced fatigue. Compr Physiol..

[3] Periard J, Racinais S (2015). Editorial: training and competing in the heat. Scand J Med Sci Sports..

[4] Rowell LB (1974). Human cardiovascular adjustments to exercise and thermal stress. Physiol Rev..

[5] Galloway SD, Maughan RJ (1997). Effects of ambient temperature on the capacity to perform prolonged cycle exercise in man. Med Sci Sports Exerc..

[6] Périard JD, Cramer MN, Chapman PG (2011). Cardiovascular strain impairs prolonged self-paced exercise in the heat. Exp Physiol..

[7] Ely MR, Cheuvront SN, Roberts WO (2007). Impact of weather on marathon-running performance. Med Sci Sports Exerc..

[8] Morante SM, Brotherhood JR. Autonomic and behavioural thermoregulation in tennis. Br J Sports Med. 2008;42:679–85. **(discussion 685)**. 10.1136/bjsm.2007.042499 18048434

[9] Mohr M, Nybo L, Grantham J (2012). Physiological responses and physical performance during football in the heat. PLoS One..

[10] Périard JD, Racinais S, Knez WL (2014). Thermal, physiological and perceptual strain mediate alterations in match-play tennis under heat stress. Br J Sports Med..

[11] Racinais S, Périard JD, Karlsen A (2015). Effect of heat and heat acclimatization on cycling time trial performance and pacing. Med Sci Sports Exerc..

[12] Nassis GP, Brito J, Dvorak J (2015). The association of environmental heat stress with performance: analysis of the 2014 FIFA World Cup Brazil. Br J Sports Med..

[13] Adolph EF (1947). Physiology of man in the desert.

[14] Strydom NB, Holdsworth LD (1968). The effects of different levels of water deficit on physiological responses during heat stress. Int Z Angew Physiol..

[15] Sawka MN, Young AJ, Francesconi RP (1985). Thermoregulatory and blood responses during exercise at graded hypohydration levels. J Appl Physiol..

[16] Montain SJ, Coyle EF (1992). Influence of graded dehydration on hyperthermia and cardiovascular drift during exercise. J Appl Physiol..

[17] González-Alonso J, Crandall CG, Johnson JM (2008). The cardiovascular challenge of exercising in the heat. J Physiol..

[18] Trangmar SJ, Chiesa ST, Stock CG (2014). Dehydration affects cerebral blood flow but not its metabolic rate for oxygen during maximal exercise in trained humans. J Physiol..

[19] Sawka MN, Leon LR, Montain SJ (2011). Integrated physiological mechanisms of exercise performance, adaptation, and maladaptation to heat stress. Compr Physiol..

[20] Bergeron MF, Bahr R, Bärtsch P (2012). International Olympic Committee consensus statement on thermoregulatory and altitude challenges for high-level athletes. Br J Sports Med..

[21] Grantham J, Cheung SS, Connes P (2010). Current knowledge on playing football in hot environments. Scand J Med Sci Sports..

[22] Bahr R, Reeser JC (2012). New guidelines are needed to manage heat stress in elite sports—the Fédération Internationale de Volleyball (FIVB) Heat Stress Monitoring Programme. Br J Sports Med..

[23] Mountjoy M, Alonso J-M, Bergeron MF (2012). Hyperthermic-related challenges in aquatics, athletics, football, tennis and triathlon. Br J Sports Med..

[24] Armstrong LE, Pandolf KB, Pandolf KB, Sawka MN, Gonzalez RR (1988). Physical training, cardiorespiratory physical fitness and exercise-heat tolerance. Physiology and environmental medicine at terrestrial extremes.

[25] Gisolfi C, Robinson S (1969). Relations between physical training, acclimatization, and heat tolerance. J Appl Physiol..

[26] Nadel ER, Pandolf KB, Roberts MF (1974). Mechanisms of thermal acclimation to exercise and heat. J Appl Physiol..

[27] Roberts MF, Wenger CB, Stolwijk JA (1977). Skin blood flow and sweating changes following exercise training and heat acclimation. J Appl Physiol..

[28] Nielsen B, Hales JR, Strange S (1993). Human circulatory and thermoregulatory adaptations with heat acclimation and exercise in a hot, dry environment. J Physiol..

[29] Lorenzo S, Halliwill JR, Sawka MN (2010). Heat acclimation improves exercise performance. J Appl Physiol..

[30] Sawka MN, Wenger CB, Pandolf KB. Thermoregulatory responses to acute exercise‐heat stress and heat acclimation. In: Fregly MJ, Blatteis CM, editors. Handbook of physiology. Section 4, environmental physiology. New York: Oxford University Press; 1996. pp. 157–85.

[31] Periard J, Racinais S, Sawka MN (2015). Adaptations and mechanisms of human heat acclimation: applications for competitive athletes and sports. Scand J Med Sci Sports..

[32] Robinson S, Turrell ES, Belding HS (1943). Rapid acclimatization to work in hot climates. Am J Physiol..

[33] Ladell WS (1951). Assessment of group acclimatization to heat and humidity. J Physiol..

[34] Flouris AD, Poirier MP, Bravi A (2014). Changes in heart rate variability during the induction and decay of heat acclimation. Eur J Appl Physiol..

[35] Pandolf KB, Burse RL, Goldman RF (1977). Role of physical fitness in heat acclimatisation, decay and reinduction. Ergonomics..

[36] Sunderland C, Morris JG, Nevill ME (2008). A heat acclimation protocol for team sports. Br J Sports Med..

[37] Garrett AT, Rehrer NJ, Patterson MJ (2011). Induction and decay of short-term heat acclimation in moderately and highly trained athletes. Sports Med..

[38] Chalmers S, Esterman A, Eston R (2014). Short-term heat acclimation training improves physical performance: a systematic review, and exploration of physiological adaptations and application for team sports. Sports Med..

[39] Karlsen A, Nybo L, Norgaard SJ (2015). Time course of natural heat acclimatization in well-trained cyclists during a 2-week training camp in the heat. Scand J Med Sci Sports..

[40] Lind AR, Bass DE (1963). Optimal exposure time for development of acclimatization to heat. Fed Proc..

[41] Houmard JA, Costill DL, Davis JA (1990). The influence of exercise intensity on heat acclimation in trained subjects. Med Sci Sports Exerc..

[42] Taylor NAS (2014). Human heat adaptation. Compr Physiol..

[43] Patterson MJ, Stocks JM, Taylor NAS (2004). Sustained and generalized extracellular fluid expansion following heat acclimation. J Physiol..

[44] Garrett AT, Goosens NG, Rehrer NJ (2009). Induction and decay of short-term heat acclimation. Eur J Appl Physiol..

[45] Racinais S, Mohr M, Buchheit M (2012). Individual responses to short-term heat acclimatisation as predictors of football performance in a hot, dry environment. Br J Sports Med..

[46] Racinais S, Buchheit M, Bilsborough J (2014). Physiological and performance responses to a training camp in the heat in professional Australian football players. Int J Sports Physiol Perform..

[47] Bean WB, Eichna LA (1943). Performance in relation to environmental temperature. Reactions of normal young men to simulated desert environments. Fed Proc..

[48] Fox RH, Goldsmith R, Hampton IF (1967). Heat acclimatization by controlled hyperthermia in hot-dry and hot-wet climates. J Appl Physiol..

[49] Eichna LW, Bean WB, William F (1945). Performance in relation to environmental temperature. Reactions of normal young men to hot, humid (simulated jungle) environment. Bull Johns Hopkins Hosp..

[50] Candas V, Libert JP, Vogt JJ (1979). Influence of air velocity and heat acclimation on human skin wettedness and sweating efficiency. J Appl Physiol..

[51] Sawka MN, Cheuvront SN, Kolka MA, Nose H, Mack GW, Imaizumi K (2003). Human adaptation to heat stress. Exercise, nutrition and environmental stress.

[52] Hellon RF, Jones RM, MacPherson RK (1956). Natural and artificial acclimatization to hot environments. J Physiol..

[53] Edholm OG (1966). The physiology of adaptation. Eugen Rev..

[54] Armstrong L, Maresh C (1991). The induction and decay of heat acclimatisation in trained athletes. Sports Med..

[55] Dresoti AO (1935). The results of some investigations into the medical aspects of deep mining on the Witwatersrand. J Chem Metal Min Soc S Afr..

[56] Lind AR, Licht S (1964). Physiologic responses to heat. Medical climatology.

[57] Weller AS, Linnane DM, Jonkman AG (2007). Quantification of the decay and re-induction of heat acclimation in dry-heat following 12 and 26 days without exposure to heat stress. Eur J Appl Physiol..

[58] Daanen HAM, Jonkman AG, Layden JD (2011). Optimising the acquisition and retention of heat acclimation. Int J Sports Med..

[59] Eichna LW, Park CR, Nelson N (1950). Thermal regulation during acclimatization in a hot, dry (desert type) environment. Am J Physiol..

[60] MacDonald DKC, Wyndham CH (1950). Heat transfer in man. J Appl Physiol..

[61] Lee D. A basis for the study of man’s reaction to tropical climates. Univ Qld Pap Dept Physiol. 1940;1:86.

[62] Buchheit M, Voss SC, Nybo L (2011). Physiological and performance adaptations to an in-season soccer camp in the heat: associations with heart rate and heart rate variability. Scand J Med Sci Sports..

[63] Buchheit M, Racinais S, Bilsborough J (2013). Adding heat to the live-high train-low altitude model: a practical insight from professional football. Br J Sports Med..

[64] Dill DB, Hall FG, Edwards HT (1938). Changes in composition of sweat during acclimatization to heat. Am J Physiol..

[65] Glaser EM (1950). Acclimatization to heat and cold. J Physiol..

[66] Sawka MN, Coyle EF (1999). Influence of body water and blood volume on thermoregulation and exercise performance in the heat. Exerc Sport Sci Rev..

[67] Watt MJ, Garnham AP, Febbraio MA (2000). Effect of acute plasma volume expansion on thermoregulation and exercise performance in the heat. Med Sci Sports Exerc..

[68] Armstrong LE, Hubbard RW, DeLuca JP (1987). Evaluation of a temperate environment test to predict heat tolerance. Eur J Appl Physiol Occup Physiol..

[69] Hue O, Antoine-Jonville S, Sara F (2007). The effect of 8 days of training in tropical environment on performance in neutral climate in swimmers. Int J Sports Med..

[70] Scoon GSM, Hopkins WG, Mayhew S (2007). Effect of post-exercise sauna bathing on the endurance performance of competitive male runners. J Sci Med Sport..

[71] Karlsen A, Racinais S, Jensen MV (2015). Heat acclimatization does not improve *V*O_2max_ or cycling performance in a cool climate in trained cyclists. Scand J Med Sci Sports..

[72] Nadel ER, Fortney SM, Wenger CB (1980). Effect of hydration state of circulatory and thermal regulations. J Appl Physiol..

[73] Fortney SM, Wenger CB, Bove JR (1984). Effect of hyperosmolality on control of blood flow and sweating. J Appl Physiol..

[74] Montain SJ, Latzka WA, Sawka MN (1995). Control of thermoregulatory sweating is altered by hydration level and exercise intensity. J Appl Physiol..

[75] González-Alonso J, Mora-Rodríguez R, Below PR (1995). Dehydration reduces cardiac output and increases systemic and cutaneous vascular resistance during exercise. J Appl Physiol..

[76] González-Alonso J, Calbet JA, Nielsen B (1998). Muscle blood flow is reduced with dehydration during prolonged exercise in humans. J Physiol..

[77] Stöhr EJ, González-Alonso J, Pearson J (2011). Dehydration reduces left ventricular filling at rest and during exercise independent of twist mechanics. J Appl Physiol..

[78] Sawka MN, Toner MM, Francesconi RP (1983). Hypohydration and exercise: effects of heat acclimation, gender, and environment. J Appl Physiol..

[79] Sawka MN (1992). Physiological consequences of hypohydration: exercise performance and thermoregulation. Med Sci Sports Exerc..

[80] González-Alonso J, Mora-Rodríguez R, Coyle EF (2000). Stroke volume during exercise: interaction of environment and hydration. Am J Physiol Heart Circ Physiol..

[81] Cheuvront SN, Kenefick RW (2014). Dehydration: physiology, assessment, and performance effects. Compr Physiol..

[82] Goulet EDB (2011). Effect of exercise-induced dehydration on time-trial exercise performance: a meta-analysis. Br J Sports Med..

[83] Goulet EDB (2013). Effect of exercise-induced dehydration on endurance performance: evaluating the impact of exercise protocols on outcomes using a meta-analytic procedure. Br J Sports Med..

[84] Cotter JD, Thornton SN, Lee JK (2014). Are we being drowned in hydration advice? Thirsty for more?. Extrem Physiol Med..

[85] Below PR, Mora-Rodríguez R, González-Alonso J (1995). Fluid and carbohydrate ingestion independently improve performance during 1 h of intense exercise. Med Sci Sports Exerc..

[86] Cheung SS, McLellan TM (1998). Heat acclimation, aerobic fitness, and hydration effects on tolerance during uncompensable heat stress. J Appl Physiol..

[87] Ebert TR, Martin DT, Bullock N (2007). Influence of hydration status on thermoregulation and cycling hill climbing. Med Sci Sports Exerc..

[88] Kenefick RW, Cheuvront SN, Palombo LJ (2010). Skin temperature modifies the impact of hypohydration on aerobic performance. J Appl Physiol..

[89] Merry TL, Ainslie PN, Cotter JD (2010). Effects of aerobic fitness on hypohydration-induced physiological strain and exercise impairment. Acta Physiol (Oxf)..

[90] Sawka MN, Cheuvront SN, Kenefick RW (2012). High skin temperature and hypohydration impair aerobic performance. Exp Physiol..

[91] Wall BA, Watson G, Peiffer JJ, et al. Current hydration guidelines are erroneous: dehydration does not impair exercise performance in the heat. Br J Sports Med. 2013. doi:10.1136/bjsports-2013-092417 10.1136/bjsports-2013-092417 24055782

[92] Cohen D (2012). The truth about sports drinks. BMJ..

[93] Adolph EF, Dill DB (1938). Observations on water metabolism in the desert. Am J Physiol..

[94] Greenleaf JE, Sargent F (1965). Voluntary dehydration in man. J Appl Physiol..

[95] Greenleaf JE, Brock PJ, Keil LC (1983). Drinking and water balance during exercise and heat acclimation. J Appl Physiol..

[96] Armstrong LE, Costill DL, Fink WJ (1985). Influence of diuretic-induced dehydration on competitive running performance. Med Sci Sports Exerc..

[97] Greenleaf JE (1992). Problem: thirst, drinking behavior, and involuntary dehydration. Med Sci Sports Exerc..

[98] Cheuvront SN, Haymes EM (2001). Ad libitum fluid intakes and thermoregulatory responses of female distance runners in three environments. J Sports Sci..

[99] Zouhal H, Groussard C, Minter G (2011). Inverse relationship between percentage body weight change and finishing time in 643 forty-two-kilometre marathon runners. Br J Sports Med..

[100] Noakes TD, Goodwin N, Rayner BL (1985). Water intoxication: a possible complication during endurance exercise. Med Sci Sports Exerc..

[101] Noakes TD (2003). Overconsumption of fluids by athletes. BMJ..

[102] Almond CSD, Shin AY, Fortescue EB (2005). Hyponatremia among runners in the Boston Marathon. N Engl J Med..

[103] Institute of Medicine (US). Dietary reference intakes for water, potassium, sodium, chloride, and sulfate. Washington, DC: The National Academies Press; 2004:73–423.

[104] Cheuvront SN, Carter R, Montain SJ (2004). Daily body mass variability and stability in active men undergoing exercise-heat stress. Int J Sport Nutr Exerc Metab..

[105] Cheuvront SN, Ely BR, Kenefick RW (2010). Biological variation and diagnostic accuracy of dehydration assessment markers. Am J Clin Nutr..

[106] Cheuvront SN, Kenefick RW, Charkoudian N (2013). Physiologic basis for understanding quantitative dehydration assessment. Am J Clin Nutr..

[107] Cheuvront SN, Montain SJ, Goodman DA (2007). Evaluation of the limits to accurate sweat loss prediction during prolonged exercise. Eur J Appl Physiol..

[108] Adams WC, Fox RH, Fry AJ (1975). Thermoregulation during marathon running in cool, moderate, and hot environments. J Appl Physiol..

[109] Bergeron MF, Armstrong LE, Maresh CM (1995). Fluid and electrolyte losses during tennis in the heat. Clin Sports Med..

[110] Bergeron MF, Maresh CM, Armstrong LE (1995). Fluid-electrolyte balance associated with tennis match play in a hot environment. Int J Sport Nutr..

[111] Shirreffs SM, Sawka MN, Stone M (2006). Water and electrolyte needs for football training and match-play. J Sports Sci..

[112] Shapiro Y, Pandolf KB, Goldman RF (1982). Predicting sweat loss response to exercise, environment and clothing. Eur J Appl Physiol Occup Physiol..

[113] Barr SI, Costill DL. Water: can the endurance athlete get too much of a good thing? J Am Diet Assoc. 1989;89:1629–35. 2681335

[114] Montain SJ, Cheuvront SN, Sawka MN. Exercise associated hyponatraemia: quantitative analysis to understand the aetiology. Br J Sports Med. 2006;40:98–105. **(discussion 98–105)**. 10.1136/bjsm.2005.018481 PMC2492017 16431994

[115] Gonzalez RR, Cheuvront SN, Montain SJ (2009). Expanded prediction equations of human sweat loss and water needs. J Appl Physiol..

[116] Gonzalez RR, Cheuvront SN, Ely BR (2012). Sweat rate prediction equations for outdoor exercise with transient solar radiation. J Appl Physiol..

[117] Jay O, Webb P (2009). Improving the prediction of sweat losses during exercise. J Appl Physiol..

[118] Costill DL (1977). Sweating: its composition and effects on body fluids. Ann N Y Acad Sci..

[119] Verde T, Shephard RJ, Corey P (1982). Sweat composition in exercise and in heat. J Appl Physiol..

[120] Bergeron MF (2008). Muscle cramps during exercise—is it fatigue or electrolyte deficit?. Curr Sports Med Rep..

[121] Casa DJ (1999). Exercise in the heat. II. Critical concepts in rehydration, exertional heat illnesses, and maximizing athletic performance. J Athl Train..

[122] von Duvillard SP, Braun WA, Markofski M (2004). Fluids and hydration in prolonged endurance performance. Nutrition..

[123] Sawka MN, Burke LM, American College of Sports Medicine (2007). American College of Sports Medicine position stand. Exercise and fluid replacement. Med Sci Sports Exerc..

[124] Bergeron MF (2003). Heat cramps: fluid and electrolyte challenges during tennis in the heat. J Sci Med Sport..

[125] Burke LM, Hawley JA, Wong SHS (2011). Carbohydrates for training and competition. J Sports Sci..

[126] Shirreffs SM, Maughan RJ (1998). Volume repletion after exercise-induced volume depletion in humans: replacement of water and sodium losses. Am J Physiol..

[127] Febbraio MA, Snow RJ, Stathis CG (1994). Effect of heat stress on muscle energy metabolism during exercise. J Appl Physiol..

[128] González-Alonso J, Calbet JA, Nielsen B (1999). Metabolic and thermodynamic responses to dehydration-induced reductions in muscle blood flow in exercising humans. J Physiol..

[129] Burke LM (2001). Nutritional needs for exercise in the heat. Comp Biochem Physiol A Mol Integr Physiol..

[130] Ivy JL, Katz AL, Cutler CL (1988). Muscle glycogen synthesis after exercise: effect of time of carbohydrate ingestion. J Appl Physiol..

[131] James L (2012). Milk protein and the restoration of fluid balance after exercise. Med Sport Sci..

[132] Beelen M, Burke LM, Gibala MJ (2010). Nutritional strategies to promote postexercise recovery. Int J Sport Nutr Exerc Metab..

[133] Pritchett K, Pritchett R (2012). Chocolate milk: a post-exercise recovery beverage for endurance sports. Med Sport Sci..

[134] Booth J, Marino F, Ward JJ (1997). Improved running performance in hot humid conditions following whole body precooling. Med Sci Sports Exerc..

[135] González-Alonso J, Teller C, Andersen SL (1999). Influence of body temperature on the development of fatigue during prolonged exercise in the heat. J Appl Physiol..

[136] Quod MJ, Martin DT, Laursen PB (2008). Practical precooling: effect on cycling time trial performance in warm conditions. J Sports Sci..

[137] Duffield R, Green R, Castle P (2010). Precooling can prevent the reduction of self-paced exercise intensity in the heat. Med Sci Sports Exerc..

[138] Ihsan M, Landers G, Brearley M (2010). Beneficial effects of ice ingestion as a precooling strategy on 40-km cycling time-trial performance. Int J Sports Physiol Perform..

[139] Ross MLR, Garvican LA, Jeacocke NA (2011). Novel precooling strategy enhances time trial cycling in the heat. Med Sci Sports Exerc..

[140] Siegel R, Maté J, Watson G (2012). Pre-cooling with ice slurry ingestion leads to similar run times to exhaustion in the heat as cold water immersion. J Sports Sci..

[141] Marsh D, Sleivert G (1999). Effect of precooling on high intensity cycling performance. Br J Sports Med..

[142] Duffield R, Marino FE (2007). Effects of pre-cooling procedures on intermittent-sprint exercise performance in warm conditions. Eur J Appl Physiol..

[143] Castle P, Mackenzie RW, Maxwell N (2011). Heat acclimation improves intermittent sprinting in the heat but additional pre-cooling offers no further ergogenic effect. J Sports Sci..

[144] Minett GM, Duffield R, Marino FE (2011). Volume-dependent response of precooling for intermittent-sprint exercise in the heat. Med Sci Sports Exerc..

[145] Brade C, Dawson B, Wallman K (2014). Effects of different precooling techniques on repeat sprint ability in team sport athletes. Eur J Sports Sci..

[146] Duffield R, Dawson B, Bishop D (2003). Effect of wearing an ice cooling jacket on repeat sprint performance in warm/humid conditions. Br J Sports Med..

[147] Cheung S, Robinson A (2004). The influence of upper-body pre-cooling on repeated sprint performance in moderate ambient temperatures. J Sports Sci..

[148] Brade C, Dawson B, Wallman K (2013). Effect of precooling and acclimation on repeat-sprint performance in heat. J Sports Sci..

[149] Sleivert GG, Cotter JD, Roberts WS (2001). The influence of whole-body vs. torso pre-cooling on physiological strain and performance of high-intensity exercise in the heat. Comp Biochem Physiol A Mol Integr Physiol..

[150] Castle PC, Macdonald AL, Philp A (2006). Precooling leg muscle improves intermittent sprint exercise performance in hot, humid conditions. J Appl Physiol..

[151] Marino FE (2002). Methods, advantages, and limitations of body cooling for exercise performance. Br J Sports Med..

[152] Quod MJ, Martin DT, Laursen PB (2006). Cooling athletes before competition in the heat: comparison of techniques and practical considerations. Sports Med..

[153] Duffield R (2008). Cooling interventions for the protection and recovery of exercise performance from exercise-induced heat stress. Med Sport Sci..

[154] Jones PR, Barton C, Morrissey D (2012). Pre-cooling for endurance exercise performance in the heat: a systematic review. BMC Med..

[155] Siegel R, Laursen PB (2012). Keeping your cool: possible mechanisms for enhanced exercise performance in the heat with internal cooling methods. Sports Med..

[156] Ross M, Abbiss C, Laursen P (2013). Precooling methods and their effects on athletic performance: a systematic review and practical applications. Sports Med..

[157] Tyler CJ, Sunderland C, Cheung SS (2015). The effect of cooling prior to and during exercise on exercise performance and capacity in the heat: a meta-analysis. Br J Sports Med..

[158] Bongers CCWG, Thijssen DHJ, Veltmeijer MTW (2015). Precooling and percooling (cooling during exercise) both improve performance in the heat: a meta-analytical review. Br J Sports Med..

[159] Morrison SA, Cheung S, Cotter JD (2014). Importance of airflow for physiologic and ergogenic effects of precooling. J Athl Train..

[160] Leeder J, Gissane C, van Someren K (2012). Cold water immersion and recovery from strenuous exercise: a meta-analysis. Br J Sports Med..

[161] DeGroot DW, Gallimore RP, Thompson SM (2013). Extremity cooling for heat stress mitigation in military and occupational settings. J Therm Biol..

[162] Versey NG, Halson SL, Dawson BT (2013). Water immersion recovery for athletes: effect on exercise performance and practical recommendations. Sports Med..

[163] Cotter JD, Sleivert GG, Roberts WS (2001). Effect of pre-cooling, with and without thigh cooling, on strain and endurance exercise performance in the heat. Comp Biochem Physiol A Mol Integr Physiol..

[164] Arngrïmsson SA, Petitt DS, Stueck MG (2004). Cooling vest worn during active warm-up improves 5-km run performance in the heat. J Appl Physiol..

[165] Cheuvront SN, Kolka MA, Cadarette BS (2003). Efficacy of intermittent, regional microclimate cooling. J Appl Physiol..

[166] Lee JKW, Shirreffs SM, Maughan RJ (2008). Cold drink ingestion improves exercise endurance capacity in the heat. Med Sci Sports Exerc..

[167] Byrne C, Owen C, Cosnefroy A (2011). Self-paced exercise performance in the heat after pre-exercise cold-fluid ingestion. J Athl Train..

[168] Lee JKW, Shirreffs SM (2007). The influence of drink temperature on thermoregulatory responses during prolonged exercise in a moderate environment. J Sports Sci..

[169] Lee JKW, Maughan RJ, Shirreffs SM (2008). The influence of serial feeding of drinks at different temperatures on thermoregulatory responses during cycling. J Sports Sci..

[170] Bain AR, Lesperance NC, Jay O (2012). Body heat storage during physical activity is lower with hot fluid ingestion under conditions that permit full evaporation. Acta Physiol (Oxf)..

[171] Morris NB, Bain AR, Cramer MN (2014). Evidence that transient changes in sudomotor output with cold and warm fluid ingestion are independently modulated by abdominal, but not oral thermoreceptors. J Appl Physiol..

[172] Siegel R, Maté J, Brearley MB (2010). Ice slurry ingestion increases core temperature capacity and running time in the heat. Med Sci Sports Exerc..

[173] Yeo ZW, Fan PWP, Nio AQX (2012). Ice slurry on outdoor running performance in heat. Int J Sports Med..

[174] Stevens CJ, Dascombe B, Boyko A (2013). Ice slurry ingestion during cycling improves Olympic distance triathlon performance in the heat. J Sports Sci..

[175] Stanley J, Leveritt M, Peake JM (2010). Thermoregulatory responses to ice-slush beverage ingestion and exercise in the heat. Eur J Appl Physiol..

[176] Duffield R, Coutts A, McCall A (2013). Pre-cooling for football training and competition in hot and humid conditions. Eur J Sports Sci..

[177] Duffield R, Steinbacher G, Fairchild TJ (2009). The use of mixed-method, part-body pre-cooling procedures for team-sport athletes training in the heat. J Strength Cond Res..

[178] Yeargin SW, Casa DJ, McClung JM (2006). Body cooling between two bouts of exercise in the heat enhances subsequent performance. J Strength Cond Res..

[179] Peiffer JJ, Abbiss CR, Watson G (2010). Effect of a 5-min cold-water immersion recovery on exercise performance in the heat. Br J Sports Med..

[180] Vaile J, O’Hagan C, Stefanovic B (2011). Effect of cold water immersion on repeated cycling performance and limb blood flow. Br J Sports Med..

[181] Hornery DJ, Papalia S, Mujika I (2005). Physiological and performance benefits of halftime cooling. J Sci Med Sport..

[182] Lee JKW, Yeo ZW, Nio AQX (2013). Cold drink attenuates heat strain during work-rest cycles. Int J Sports Med..

[183] Armstrong LE, Casa DJ, American College of Sports Medicine (2007). American College of Sports Medicine position stand. Exertional heat illness during training and competition. Med Sci Sports Exerc..

[184] Budd GM (2008). Wet-bulb globe temperature (WBGT)-its history and its limitations. J Sci Med Sport..

[185] Gonzalez RR (1995). Biophysics of heat exchange and clothing: applications to sports physiology. Med Exerc Nutr Health..

[186] Aughey RJ, Goodman CA, Mckenna MJ (2014). Greater chance of high core temperatures with modified pacing strategy during team sport in the heat. J Sci Med Sport..

[187] Gosling CM, Gabbe BJ, McGivern J (2008). The incidence of heat casualties in sprint triathlon: the tale of two Melbourne race events. J Sci Med Sport..

[188] Sartor F, Snacken R, Demuth C (1995). Temperature, ambient ozone levels, and mortality during summer 1994, in Belgium. Environ Res..

[189] Byrne C, Lee JKW, Chew SAN (2006). Continuous thermoregulatory responses to mass-participation distance running in heat. Med Sci Sports Exerc..

[190] Roberts WO (2006). Exertional heat stroke during a cool weather marathon: a case study. Med Sci Sports Exerc..

[191] Sonna LA, Wenger CB, Flinn S (2004). Exertional heat injury and gene expression changes: a DNA microarray analysis study. J Appl Physiol..

[192] Roberts WO (2010). Determining a “do not start” temperature for a marathon on the basis of adverse outcomes. Med Sci Sports Exerc..

